# Identifying the Underlying Factors Associated With Patients’ Attitudes Toward Antidepressants: Qualitative and Quantitative Analysis of Patient Drug Reviews

**DOI:** 10.2196/10726

**Published:** 2018-09-30

**Authors:** Maryam Zolnoori, Kin Wah Fung, Paul Fontelo, Hadi Kharrazi, Anthony Faiola, Yi Shuan Shirley Wu, Virginia Stoffel, Timothy Patrick

**Affiliations:** 1 Lister Hill National Center for Biomedical Communications National Library of Medicine National Institutes of Health Bethesda, MD United States; 2 Department of Health Informatics and Administration College of Health Sciences University of Wisconsin-Milwaukee Milwaukee, WI United States; 3 Section of Medical Informatics Department of Health Science Research Mayo Clinic Rochester, MN United States; 4 Center for Population Health IT Department of Health Policy and Management Johns Hopkins Bloomberg School of Public Health, Johns Hopkins University Baltimore, MD United States; 5 Biomedical and Health Information Sciences College of Applied Health Sciences University of Illinois at Chicago Chicago, IL United States; 6 UNC Eshelman School of Pharmacy University of North Carolina Chapel Hill, NC United States; 7 Department of Occupational Science & Technology College of Health Sciences University of Wisconsin-Milwaukee Milwaukee, WI United States; 8 Industrial and Manufacturing Engineering College of Engineering & Applied Science University of Wisconsin-Milwaukee Milwaukee, WI United States

**Keywords:** medication adherence, attitude, perception, antidepressive agents, patient-centered care, chronic disease, depression, community networks, internet, social media, data mining, framework method

## Abstract

**Background:**

Nonadherence to antidepressants is a major obstacle to deriving antidepressants’ therapeutic benefits, resulting in significant burdens on the individuals and the health care system. Several studies have shown that nonadherence is weakly associated with personal and clinical variables but strongly associated with patients’ beliefs and attitudes toward medications. Patients’ drug review posts in online health care communities might provide a significant insight into patients’ attitude toward antidepressants and could be used to address the challenges of self-report methods such as patients’ recruitment.

**Objective:**

The aim of this study was to use patient-generated data to identify factors affecting the patient’s attitude toward 4 antidepressants drugs (sertraline [Zoloft], escitalopram [Lexapro], duloxetine [Cymbalta], and venlafaxine [Effexor XR]), which in turn, is a strong determinant of treatment nonadherence. We hypothesized that clinical variables (drug effectiveness; adverse drug reactions, ADRs; perceived distress from ADRs, ADR-PD; and duration of treatment) and personal variables (age, gender, and patients’ knowledge about medications) are associated with patients’ attitude toward antidepressants, and experience of ADRs and drug ineffectiveness are strongly associated with negative attitude.

**Methods:**

We used both qualitative and quantitative methods to analyze the dataset. Patients’ drug reviews were randomly selected from a health care forum called *askapatient.* The Framework method was used to build the analytical framework containing the themes for developing structured data from the qualitative drug reviews. Then, 4 annotators coded the drug reviews at the sentence level using the analytical framework. After managing missing values, we used chi-square and ordinal logistic regression to test and model the association between variables and attitude.

**Results:**

A total of 892 reviews posted between February 2001 and September 2016 were analyzed. Most of the patients were females (680/892, 76.2%) and aged less than 40 years (540/892, 60.5%). Patient attitude was significantly (*P*<.001) associated with experience of ADRs, ADR-PD, drug effectiveness, perceived lack of knowledge, experience of withdrawal, and duration of usage, whereas oth age (F_4,874_=0.72, *P*=.58) and gender (χ^2^_4_=2.7, *P*=.21) were not found to be associated with patient attitudes. Moreover, modeling the relationship between variables and attitudes showed that drug effectiveness and perceived distress from adverse drug reactions were the 2 most significant factors affecting patients’ attitude toward antidepressants.

**Conclusions:**

Patients’ self-report experiences of medications in online health care communities can provide a direct insight into the underlying factors associated with patients’ perceptions and attitudes toward antidepressants. However, it cannot be used as a replacement for self-report methods because of the lack of information for some of the variables, colloquial language, and the unstructured format of the reports.

## Introduction

### Background

The prevalence of use of antidepressants among Americans increased from 7.7% in the years 1999 to 2002 to 12.7% in 2011 to 2014 [1], with the global market estimated at US $11.6 billion in 2017 [2]. The therapeutic benefits of antidepressants depend on adherence to prescribed regimen; however, between 30% and 68% of patients are nonadherent [3], leading to increased risks of depression relapse, emergency visits, low quality of life, and significant burdens on the individual and health care system [4].

Several studies indicate that nonadherence is weakly associated with personal attributes and clinical variables, but it is strongly associated with patients’ beliefs and attitudes toward medication [3,5,6]. Identifying the key dimensions of patients’ attitudes toward antidepressants is a challenging task [7]. Although self-report scales for measuring patients’ attitude toward antidepressants are well validated, they are associated with some methodological difficulties (eg, missing factors influencing attitude, sampling bias, and patients’ reluctance to reveal personal information). On the other hand, generic scales such as the Beliefs about Medicines Questionnaire [8] and Drug Attitude Inventory [9] that are widely used in many patient groups are not specifically designed to evaluate patients’ attitudes toward antidepressants.

Online health care communities have provided patients with a unique platform to report their experiences freely and express their main concerns and perceptions about their treatments. This information may not be collected by traditional self-report methods such as questionnaires, interviews, or physician assessments [10-12]. A public opinion survey found that 30% of Americans actively participate in creating health-related knowledge in online health care forums [13]. This rate is higher among patients with mental disorders, possibly due to stigma against them [14]. With the growing emphasis on patient-centered care, the ability to directly measure individuals’ attitudes toward medications from their reviews in social media may increase early detection of factors that contribute to nonadherence and negative outcomes [15]. To the extent of our knowledge, no study has focused on identifying underlying factors influencing attitude toward antidepressant treatment as reported by patients in online health care forums.

### Contributions

The premise of this study is that patients’ self-reports of their experiences with antidepressants therapy on drug review forums may constitute a reliable source to uncover various dimensions of attitude toward these medications.

In this study, we utilized the online health care forum called *askapatient* to identify underlying factors associated with attitudes toward antidepressants. We used a novel mixed-method approach to generate structured data from unstructured text, evaluate the association between attitude and both personal and clinical variables, and model the relationship between the variables and patients’ attitudes toward antidepressants. To achieve the latter, we identified clinical and personal factors from literature that have shown to affect attitude toward psychiatric medications and then used these factors for designing an initial framework of analysis for patients’ drug reviews.

We hypothesize that clinical variables including drug effectiveness, experience of adverse drug reactions (ADRs), perceived distress from ADRs (ADR-PD), and duration of treatment are associated with patients’ attitudes toward antidepressants. We also hypothesize that drug effectiveness and presence of adverse effects are the most important factors affecting patients’ attitudes toward medications. Furthermore, we hypothesize that personal variables including age, gender, and patients’ lack of knowledge about medications are associated with the patients’ attitudes toward antidepressants.

## Methods

### Summary of the Method

**Figure 1:**
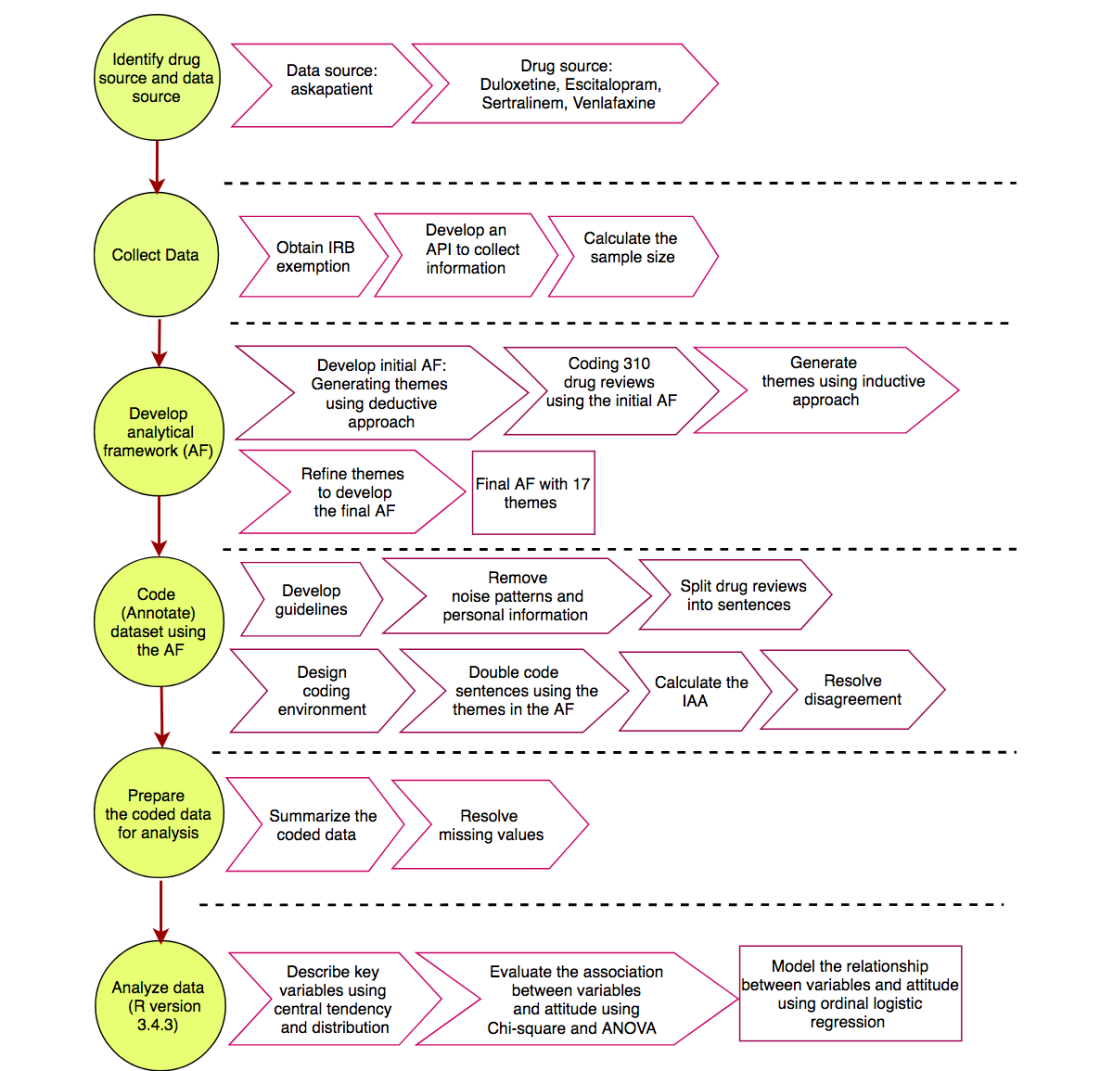
A summary of the research methodology of the study. IAA: interannotator agreement; ANOVA: analysis of variance; API: application programming interface; IRB: institutional review board.

The methodology of this study is composed of multiple phases. We first generated structured data from unstructured patients’ review using the analytical framework method. Then, we used the structured data to test the hypotheses and model the relationship between variables and attitude. Figure 1 shows the summary of the methodology for this study.

### Drug Sources and Data Source

The data source of this study is a health care forum called *askapatient*. This health care forum collects patients’ experiences for a wide-range of medications, along with the patients’ age, gender, reason for drug prescription, and duration of usage. Patients can also rate their satisfactions with the drugs through a Likert scale from 1 (not satisfied) to 5 (very satisfied). For the purpose of this study, we considered the patients’ satisfaction with the drugs as their overall attitudes toward the medications.

Patient satisfaction in several studies has been characterized by patients’ beliefs and attitudes [16,17]. In addition, the Likert scale is equivalent to the scales used by attitude studies to present outcome of self-report scales such as Drug Attitude Inventory and Antidepressant Compliance Questionnaire.

The drug sources for this study are sertraline and escitalopram from the selective serotonin reuptake inhibitor (SSRI) class, and venlafaxine and duloxetine from the serotonin-norepinephrine reuptake inhibitor (SNRI) class. We chose these drugs because they are associated with a wide range of ADRs and withdrawal symptoms (WDs) that might affect the patients’ attitudes toward the drugs and because they are among the most commonly prescribed antidepressants [18].

### Data Collection

We randomly collected 892 drug reviews for the 4 antidepressants that were posted between February 2001 and September 2016. The sample size was calculated using the formula introduced by Barlett et al [19] for categorical data. We applied stratified sampling procedure so that the proportion of patients in each attitude group (1-5) was an approximate of the full population.

As this health care forum does not have an application programming interface, we developed a Web crawler system to collect all the drug reviews from the forum. University of Wisconsin-Milwaukee’s institutional review board exempted this study as the study data are publicly available and no patient consent was required.

### Developing the Analytical Framework

We used the Framework Method to summarize patients’ experience with medications. The Framework Method is a flexible tool that uses inductive, deductive, or hybrid approaches (combination of inductive and deductive approaches) to generate themes for developing highly structured data from qualitative data [20]. In the deductive approach, themes are generated using literature, whereas in the inductive approach, themes are generated using open coding. In this study, we adopted a hybrid method that combines both inductive and deductive approaches for generating themes to analyze patients’ reviews in online healthcare communities.

### Generating Themes Using Deductive Approach

We conducted a comprehensive review of the literature to identify pertinent factors affecting patients’ attitude toward antidepressants. The identified factors were categorized into 5 categories: pharmaceutical treatment, health care system, psychosocial, patient-related, and depression-related factors. These factors were used as the themes to construct a preliminary analytical framework for data analysis. Table 1 shows the categories of the themes and the themes in each category. The details of the themes are available in [21].

### Generating Themes Using Inductive Approach (Open Coding)

A total of 310 drug reviews were randomly selected for analysis using the preliminary analytical framework. Passages of drug reviews that could not be covered by the preliminary analytical framework were discussed in our regular team meeting for generating new themes. The identified themes reflected patients’ experiences with the medications. Using this approach, 8 new themes were generated: WDs, perceived distress from WDs, intentional withdrawal (discontinuation), unintentional withdrawal (missing dose, running out of medication), patient recommendations to others, overall attitude toward medications, problem with financial support, and problem with social support.

### Developing the Final Analytical Framework

To reduce the complexity of the data analysis, themes that covered less than 5% of the drug reviews were eliminated or merged with other themes. For example, *affordability* and *partner support* were excluded, and *general concern and necessity* were merged with *overall attitude toward drug*. Themes that were conceptually related to other themes but difficult to distinguish were merged in the final analytical framework. For example, *perceived necessity* and *perceived effectiveness* were merged. We also removed patients’ general attitudes toward medications because they were strongly correlated with patients’ rating (satisfactions) for the drugs. Table 2 includes definitions of the themes and subthemes used in the final analytical framework with examples of patients’ reviews for the drugs.

### Analysis of Dataset Using the Analytical Framework

This phase consisted of the following 2 main steps: (1) data preprocessing and (2) annotating the dataset using the themes in the analytical framework.

#### Data Preprocessing

The majority of drug review posts were composed of multiple sentences, each of which covers various aspects of patients’ experiences with drugs. To improve accuracy of data analysis and reduce the observational error, we set the unit of analysis at the sentence level. To split the reviews into sentences, we first addressed the grammatical and punctuation errors in colloquial language using regular expression, and then we applied the Natural Language Toolkit [22] to split reviews into separate sentences.

**Table 1:** Factors affecting patients’ attitudes toward antidepressants (identified by a comprehensive review of the literature).

Category	Factors in each category
Pharmacological treatment factors	Perceived effectiveness Perceived necessity Perceived concern Adverse drug reaction Perceived distress from the adverse effect
Health care system factors	Patient-provider relationship Health care setting Affordability of the treatment
Social-cognitive and psychological factors	Stigma and cultural related factors Partners’ support
Patient-related factors	General concern and necessity Knowledge about pharmacological aspects of medication Sociodemographic factors Educational level
Depression factors	Depression severity, type, and duration Patient insight about depression

#### Annotating Sentences Using the Themes in the Analytical Framework

All drug reviews in the sample were annotated using the analytical framework at sentence level. Four annotators with health background participated in the data annotation process. All sentences were double coded. The defined items in the framework were not mutually exclusive. In other words, a sentence may be annotated as more than 1 individual theme. For example, this sentence “It really helped me, however I suffered from side effects.” was annotated as both as “effectiveness” and “perceived distress from adverse drug reaction.”

#### Calculating Interannotator Agreement

We used Cohen kappa to calculate interannotator agreement (IAA) [23]. The overall kappa score for the entire dataset was .75 with the highest value for perceived distress from ADR-low (.89) and the lowest for patient-physician interaction-positive (.50). To resolve the disagreement, instances of disagreement were reviewed and discussed by the same annotators who annotated the respective document earlier. For a specific item, annotation was added or removed if they were marked by any of the annotators, given that they both agreed on the decision. Otherwise, the sentences were labeled as “others.”

**Table 2:** Themes and subthemes used in the final analytical framework with examples.

Themes	Description	Example
Adverse drug reactions-presence or absence	If the patient explicitly reported experiencing ADRs^a^ associated with the drug consumption.	“Effexor XR gave me horrible nightmares and I kept waking up.”
Perceived distress from ADRs-high	If the patient explicitly mentioned that they suffered from ADRs, used any qualifiers indicating severe ADRs, or indicated functional problems such as limitation in daily functioning because of ADRs.	“The side effects are intolerable.”
Perceived distress from ADRs-low	If the patient explicitly mentioned that the ADRs were tolerable and/or used qualifiers indicating mildness/transience persistency of ADRs.	“Any side effects were, for me, tolerable compared to the benefits.”
Perceived distress from ADRs-medium	If the perceived distress form ADR cannot be labeled as high or low, then it is medium.	“I suffered from headache.”
Withdrawal symptoms-presence or absence	If the patient explicitly reported experiencing any sign/symptoms associated with the process of dosage reduction or drug discontinuation.	“I weaned slowly and I feel nauseous a lot.”
WD^b^-perceived distress-high	If the patients explicitly mentioned they suffered from withdrawal symptoms, functional problems associated with the WDs, or they used qualifiers indicating the severity of a specific WD.	“The withdrawal symptoms are horrible**.”**
WD-perceived distress-low	If the patient explicitly mentioned that the WDs were tolerable or used qualifiers indicting mildness/transience of WDs.	“Withdrawal was fine; When I stopped the drug, I had mild dizziness.”
Perceived distress from WDs-medium	If the perceived distress form WD cannot be labeled as high or low, then it is medium.	“When I tried to stop the drug, I had some dizziness.”
Drug effectiveness-effectiveness	A drug is effective if the patient reported that depression symptoms improved or resolved after drug consumption.	“For the first few weeks it helped me feel better.”
Drug effectiveness-ineffectiveness	A drug is ineffective if the patient reported that depression symptoms became worse or stayed the same.	“It did not help me at all.”
Patient-physician interaction-positive	A patient-physician interaction is positive, if the patient expressed their satisfaction from communications with clinicians.	“Success with these meds truly depends on staying in touch with your physician.”
Patient-physician interaction-negative	A patient-physician interaction is negative, if the patient expressed their dissatisfaction from communications with clinicians.	“Doctors do not understand the crazy side effects of starting this class of drugs.”
Lack of knowledge	If the patient complained that they did not receive sufficient information about ADRs or WDs of the drugs and the mechanism of its management.	“No one informed me of the withdrawal nightmare.”
Experience of WD-unintentional	If the patient explicitly mentioned that they forgot to take medication (missing dosages) or ran out of medication, the discontinuation is unintentional.	“When I miss a day I feel very spaced out, thick, groggy, sad.”
Experience of WD-intentional	If the patient explicitly mentioned that they stopped (discontinue) the medication or they are in the process of discontinuation (weaning off or tapering off).	“I had to stop taking it.”

^a^ADRs: adverse drug reactions.

^b^WD: withdrawal symptoms.

### Preparing the Dataset for Analysis

#### Summarizing the Dataset

As data analysis was conducted at sentence level, an individual patient’s review may be annotated several times for availability of a theme. To summarize annotation for each patient’s review, multiple expressions of a theme for a single review were reduced to 1. If for a single review, perceived distress from ADRs or WDs was annotated as both high and low, we considered perceived distress-high as the representative of that single review. If a single review was annotated for both effectiveness and ineffectiveness, we retained both expressions of themes.

#### Strategy for Handling Missing Values

Strategies for handling missing values are composed of the following steps:

##### Elimination of the Missing Values

To handle the missing values, we first eliminated all the drug reviews with no text. Any review that did not provide information for the themes (variables) in the analytical framework was also removed from the dataset. The variable “patient-physician interaction” was also removed because of low IAA (50%) and high number of missing values.

##### Imputation of the Missing Values

To handle the rest of the missing values, we adopted different imputation methods depending on the nature of the missing values for each variable. For the variables “age,” “gender,” and “duration of usage,” missing values were imputed under the assumption of “missing completely at random”; that is, the missing values are a random sample of the complete data. The variable “age” was imputed by mean, “gender” by mode, and “duration of usage” by median. For “drug effectiveness,” the missing data were imputed under the “missing at random assumption.” Under this assumption, the missing values were modeled as a function of other variables in the dataset. The k-nearest neighborhood was used for estimating the missing values for this variable. For the rest of the variables, an individual drug review was annotated for the availability of the expression of that value (themes); otherwise, it was labeled as absent. Therefore, the variables did not include any missing values.

### Data Analysis Methods

All analysis was conducted using R version 3.4.3. Descriptive statistics of central tendency and distribution were used to describe the key variables for the sample. Chi-square statistics were used to assess categorical associations. Analysis of variance was applied to study a mix of continuous and categorical variables. Ordinal logistic regression was used to model the relationship between the independent variables and attitude (dependent variable). Alpha value was set at .05 (two-tailed) for assessing statistical significance.

## Results

### Data Source Characteristics

Table 3 summarizes the characteristics of the data sample. The drug reviews were posted between February 2001 and February 2016. A total of 5 of the drug reviews did not have any text and were removed from the dataset. Approximately half of the patients were satisfied with the drugs specified in this study, indicating that unsatisfied patients are not dominant in the sample. The majority of the patients were female (680/892, 76.2%), which is in accordance with the report published by the Centers for Disease Control and Prevention showing that 2 times as many women use antidepressants as men [1]. Approximately two-thirds of the patients were aged less than 40 years, implying that younger patients are more willing to report their experiences with medications in online health care forums. Duration of medication usage ranged from 1 day to 20 years. Patients reporting an experience after 1 day might indicate concerns about drug mechanisms. Assessing duration of usage revealed that 37% of the reviews were made by patients in acute phase of depression treatment, 28% were reported by patients in the continuation phase of treatment, and 34% were reported by patients in the maintenance phase of treatment. This information indicates that drug reviews were almost evenly distributed between 3 phases of antidepressant treatment.

### Frequency of the Variables

Table 4 shows the frequency of the variables in the sample. More than 90% of the patients reported that they experienced ADRs associated with antidepressants, whereas more than half reported they were distressed by the ADRs. Almost two-thirds of the patients reported that the antidepressants were effective in treating depression symptoms and improving functional abilities. Almost 30% of the patients reported intentional drug discontinuation, whereas only 5% reported unintentional drug discontinuation. Less than 10% of the patients provided any information on their perceived experience of communication with health care providers; therefore, we removed the variable patient-physician interaction from the data analysis.

### Association Between Attitude and Variables

Analyses of associations were conducted to determine whether patients’ attitudes are associated with any of personal and clinical variables specified in the dataset. The variables “experience of ADRs” (χ^2^_4_=31.1, *P*<.001), “ADR-PD” (χ^2^_8_=231.6, *P*<.001), “drug effectiveness” (χ^2^_8_=548.5, *P*<.001), “complaint about the lack of knowledge” (χ^2^_4_=59.4, *P*<.001), “experience of withdrawal” (intentional and/or unintentional; χ^2^_4_=55.6, *P*=<.001), and “duration of usage” (*F*_4,874_=43.66, *P*<.001) were strongly associated with patients’ attitude toward medications. However, age (*F*_4,874_=0.72, *P*=.58) and gender (χ^2^_4_=2.7, *P*=.21) were not associated with the patient attitude toward the drugs. In summary, the results support the hypotheses that clinical variables (experience of ADRs, perceived distress of ADRs, and drug effectiveness) and personal variable (complaint about the lack of knowledge about medications) were related to patients’ attitude toward antidepressants. However, the results did not support the hypotheses that age and gender were associated with patients’ attitude toward antidepressants.

**Table 3:** Sample statistics for reviews posted between February 2001 and September 2016 (N=892).

Sample statistics		Sample
Number of reviews with text, n (%)		887 (99.4)
Number of reviews provided information for the variables of this study, n (%)		879 (98.5)
**Attitude^a^, n (%)**		
	Rated as 1	195 (22.2)
	Rated as 2	104 (11.8)
	Rated as 3	152 (17.3)
	Rated as 4	209 (23.8)
	Rated as 5	219 (24.9)
**Gender, n (%)**		
	Female	680 (76.2)
	Male	212 (23.8)
**Age (years)**		
	Mean (SD)	37 (12.03)
	Median (range)	35 (14-83)
**Age categories (years), n (%)**		
	<20	49 (5.6)
	20-29	242 (27.5)
	30-39	249 (28.3)
	40-49	200 (22.7)
	50-59	106 (12.1)
	≤60	33 (3.8)
**Duration of usage (months)**		
	Mean (SD)	18 (31.7)
	Median (range)	5 (1 day-240 months [20 years])
**Duration of usage categories, n (%)**		
	<1 month	215 (24.5)
	1 to <3 months	116 (13.2)
	3 to <6 months	120 (13.6)
	6 months to <1 year	125 (14.2)
	1 to <2 years	82 (9.3)
	2 to <5 years	128 (14.6)
	5 to <10 years	66 (7.5)
	≥10 years	27 (3.1)

^a^Average of rating: 3.16.

**Table 4:** Frequency of variables in the dataset.

Variables		Frequency, n (%)
**Adverse drug reactions (ADR)**		
	Presence	823 (93.6)
	Absence	56 (6.4)
**ADR-perceived distress**		
	High	483(54.9)
	Medium	230 (26.2)
	Low	166(18.9)
**Drug effectiveness**		
	Effectiveness	524 (59.6)
	Effectiveness-ineffectiveness	120 (13.6)
	Ineffectiveness	235 (26.8)
**Patient-physician interaction**		
	Negative	47 (5.3)
	Positive	62 (7.1)
	Negative-positive	4 (0.5)
	Missing value	766 (87.1)
**Complain of the lack of knowledge**		
	Presence	60 (6.8)
	Absence	819 (93.2)
**Experience of withdrawal (intentional and/or unintentional)**		
	No experience	508 (57.8)
	Experience	371 (42.2)
**Unintentional withdrawal**		
	No report	831 (94.5)
	Reported	48 (5.5)
**Intentional withdrawal**		
	No report	639 (72.7)
	Reported	240 (27.3)

### Modeling the Relationship Between Variables and Attitude

The relationship between attitude and the variables “experience of ADR,” “perceived distress of ADRs (ADR-PD),” “drug effectiveness,” “experience of WD,” “duration of usage,” and “complaint about the lack of knowledge” were modeled using ordinal logistic regression. The equation for the model is as follows:

Attitude ~ Experience of ADR + ADR_Perceived Distress + Effectiveness + Experience of WD + Duration of Usage + Lack of Knowledge

The variables “age” and “gender” were excluded from this model because they were not significantly associated with the patients’ attitudes toward antidepressants. Table 5 shows the coefficient, the SE, and the *P* value for the outcome variables for this model.

The coefficient for the variables in the predictive model shows that *perceived ineffectiveness* decreases the log odds of patients’ attitude toward antidepressants by 3.97 compared with *perceived effectiveness*. For ADR-PD, having ADR-PD low versus high changes the log odds by 1.93. For duration of treatment, for every additional day of treatment, the log odd of attitude increases by 0.0002. For the variables “experience of withdrawal,” “complaint of the lack of knowledge,” and “experience of ADR,” for every unit change in this variable (absence vs presence), the log odds of attitude changes by −0.7, −0.4, and −0.5, respectively. The results support the hypothesis that drug effectiveness is the most important factor affecting attitude toward antidepressants. Experience of ADRs compared with perceived distress of ADRs is a less important factor. A patient’s attitude toward antidepressants is influenced more by perceived distress received from ADRs than experience of ADRs.

**Table 5:** Coefficients of the variables in the predictive model.

Variable		Coefficient	SE	*P* value
Experience of ADR^a^		−0.51	1.17e-01	<.001
ADR-PD^b^-low		1.94	1.87e-01	<.001
ADR-PD-medium		0.81	1.58e-01	<.001
**Base (ADR-PD-high)**				
	Effectiveness-ineffectiveness	−0.87	1.94e-01	<.001
	Ineffectiveness	−3.98	2.12e-01	<.001
**Base (effectiveness)**				
	Experience of withdrawal	−0.7	1.38e-01	<.001
	Complaint of the lack of knowledge	−0.43	3.22e-01	.17
	Duration	0.00025	8.44e-05	.002

^a^ADR: adverse drug reactions.

^b^ADR-PD: perceived distress from ADRs.

## Discussion

### Principal Findings

In this study, we explored usability of the patients’ self-report experience in an online health care forum to generate hypotheses concerning the association between personal and clinical variables with attitude. We used a mixed-method approach to generate structured data from unstructured text, evaluate the hypotheses, and model the relationship between attitude and the identified variables. Our findings showed that in line with the literature, *drug ineffectiveness* [24,25], *experience of ADR* [26-28], *lack of knowledge* [29,30], and *duration of usage* were associated with negative attitude toward antidepressants. Association between variables “patient-physician interaction” and attitude was not tested in this study because of low IAA and high rate of missing values.

The demographic variables “age” and “gender” are not associated with levels of attitude. Our findings for age and gender are in agreement with the findings of the studies conducted by Jacob et al [31], Murata et al [27], and Ng at al [28]. However, these findings are in contrary to the findings of the study conducted by Prins et al [32].

Data analysis of this study showed that drug review posts in social media provide significant insight into patients’ perceptions and attitudes toward antidepressants as well as the pharmacological factors. However, they may not provide significant insights into patients’ intentional nonadherence because the key factor in defining adherence is the patient’s agreement with the health care provider’s treatment plan. Thus, further inquiry may be needed to determine whether the antidepressant discontinuation was in consultation with clinicians.

### Implications of the Study

The findings of this study have significant implications for developing clinical interventions aiming to improve patient attitude and adherence toward medications. One major finding is that patients’ lack of knowledge about drug mechanism and potential adverse effects may negatively influence patient attitude toward antidepressants. Prescribers are therefore well advised to inform patients about the potential risks of antidepressants and assist them in achieving realistic expectation of the treatment. Another major implication of this study is that perceived distress received from ADRs and WDs are significant predictors for patients’ attitude and in turn, medication adherence. Clinicians could encourage patients to record adverse effects and their impacts on daily functioning to identify the patients’ actual experience with the drugs. This information may help clinicians tailor interventions to improve patients’ perception of medication and consequently adherence to the antidepressant treatment. Moreover, because patient attitude toward antidepressants are shaped by perceived drug effectiveness, and antidepressants’ full effects are not seen for typically 4 to 6 weeks, clinicians should track patients’ response and encourage them to complete an adequate trial. Several studies have shown that physician support can significantly improve patient attitude and adherence toward medications.

The dataset generated using the analytical framework can be used for designing a patient-driven self-report scale for measuring patient attitude toward antidepressants. This dataset shows how patients express their concerns, complaints, and feelings about pharmacological effects of antidepressants. Ultimately, using this colloquial language in designing self-report scale may reduce the risk of patient misinterpreting the questions.

The methodology of data analysis and the analytical framework developed in this study have significant implications for data analysis of patient experiences with pharmacologic agents collected in other health care forums or reported through patient portals.

### Limitations

Several study limitations are worth noting:

1. Although patients’ self-report experience of the medications provides a significant insight into underlying factors affecting attitudes toward medications, self-reported information is not a rich source of patients’ perceptions toward health care providers, general perceived need and concerns for medications, and perceived social support. In contrast, the Antidepressant Compliance Questionnaire and the Beliefs about Medicines Questionnaire scales measure these factors using a self-reporting method. Overall, drug reviews in online health care forums cannot be used as a replacement for self-report scales measuring patients’ attitudes toward antidepressants. However, they can serve as a supplementary source for measuring patients’ attitude toward antidepressants.
2. This dataset spans from 2001 to 2016. Although this dataset provides a general picture of the underlying factors affecting patients’ attitudes toward antidepressants, it does not reflect the changes in prescribing guidelines for antidepressants during the 15 years. Changes in the antidepressants’ dosages can affect patients’ experiences with ADRs and perceived effectiveness of the drugs. Future studies can compare the trend of patients’ attitudes toward antidepressants and changes in prescribing guidelines.
3. The sample of this study includes a combination of patients’ experience in acute, continuous, and maintenance phases of antidepressants treatment, ranging from 1 day to 20 years. Although combining and analyzing patients’ experiences in different phases of treatment can provide an overall insight into the factors affecting patients’ attitude to antidepressants, it does not provide precise information about the underlying factors affecting each specific phase. Future studies may focus on a specific phase of antidepressant treatment to identify the underlying factors and compare the findings between the phases.
4. There is the concern that findings of the study may mostly reflect patients’ experiences at the maintenance phase of the antidepressant treatment. However, the statistics on the sample show that 37% of patients reported duration of treatment of less than 3 months, 28% reported between 3 months and less than 1 year, and 33% of the patients reported less than 1 year. This statistic indicates that the sample is a good representation of patients in different phases of treatment and long-time users are not dominant. Although the patients’ experiences with the medications are different and patients with longer experience may provide more information about the antidepressant, the majority of the patients provided information for the variables (themes) used for data analysis of this study.
5. Although the result of this study may be generalized to other antidepressants from the SSRI and SNRI class, it may not be generalized to other classes, such as the tricyclic antidepressants or the norepinephrine and dopamine reuptake inhibitors, as the ADRs and WDs may be different.
6. Since we collected the data from a single health care forum, there is a risk that the findings are not representative of patients in other online health care communities. The review posts in an online health care forum may also not be a representative source for all demographic groups. Some minorities, poor, or elderly patients may lack the literacy, access, or skill to report their experiences in an English-speaking online health care forum.
7. Although online health care forums provide a platform for patients to report their perceptions and attitudes toward medication freely, the risk of inaccurate reporting and false information cannot be eliminated.
8. Even though the dataset is double coded, there is the possibility that annotators did not interpret a sentence correctly and therefore assigned it to a wrong theme.
9. Although patients in the health care forum reported their major concerns about medications, the forum does not prompt patients to report their experience with withdrawal or drug effectiveness. Therefore, some patients may not report their experiences for the variables, causing bias in data analysis.
10. Finally, there is a concern for negative response bias as the patients voluntarily choose to share their experience online. However, almost 50% of patients in this study were satisfied or highly satisfied with their antidepressant medications, compared with only 35% of patients who were dissatisfied or highly dissatisfied. In addition, nearly half of the reviewers used the antidepressants for more than a year. Both findings suggest that the reviewers were not the most dissatisfied patients using antidepressants in this health care forum.

### Future Work

Several future research directions are suggested by the results. First, the analytical framework developed in this study may be applied for analysis of patient self-reported experiences for other types of medications. Analyzing data using this framework can assist researchers in identifying underlying factors associated with patients’ attitudes and perceptions as well as medication discontinuation. Another area for inquiry is to identify and normalize patients’ expressions of ADRs and WDs of the antidepressants, then measure their associations with patients’ attitude. Current studies measuring adverse effects associated with antidepressants use the Antidepressants Side-Effect Checklist, which does not include a comprehensive list of the ADRs. Exacting the ADRs from patients’ experiences may address the limitation of the self-report scales. Finally, the dataset generated in this study can be used for training text mining algorithm and machine learning systems to automatically extract from patients’ expressions, the wide range of information related to adverse effects of drugs.

### Conclusions

In this study, we showed that self-report experiences of a drug by patients in an online health care forum could provide a unique insight into identifying underlying factors associated with patients’ perceptions and attitudes to antidepressants. However, it cannot be used as an alternative for self-report scales and interview methods due to its lack of information for some of the variables, colloquial language, and the unstructured format of the data. The data analysis also showed that drug reviews might not be a reliable source for predicting patients’ intentional nonadherence behavior. Further inquiry may be needed to determine whether the medication discontinuation was in consultation with clinicians or not.

## Abbreviations

ADR: adverse drug reaction
ADR-PD: perceived distress from ADRs
IAA: interannotator agreement
SSRI: selective serotonin reuptake inhibitor
SNRI: serotonin-norepinephrine reuptake inhibitor
WD: withdrawal symptom
